# A dinuclear ruthenium(II) complex as turn-on luminescent probe for hypochlorous acid and its application for in vivo imaging

**DOI:** 10.1038/srep29065

**Published:** 2016-06-30

**Authors:** Zonglun Liu, Kuo Gao, Beng Wang, Hui Yan, Panfei Xing, Chongmin Zhong, Yongqian Xu, Hongjuan Li, Jianxin Chen, Wei Wang, Shiguo Sun

**Affiliations:** 1 College of Science, Northwest A&F University, Yangling, Shaanxi, 712100, China; 2 Beijing University of Chinese Medicine, No. 11 Beisanhuandonglu, Chaoyang District, Beijing, 100029, China

## Abstract

A dinuclear ruthenium(II) complex Ruazo was designed and synthesized, in which oxidative cyclization of the azo and *o*-amino group was employed for the detection of hypochlorous acid (HClO) in aqueous solution. The non-emissive Ruazo formed highly luminescent triazole-ruthenium(II) complex in presence of HClO and successfully imaged HClO in living cell and living mouse.

Reactive nitrogen species (RNS) and reactive oxygen species (ROS) mediate a wide variety of biological events like aging^1^, pathogen response^2^ and immunity^3^. ROS, such as HClO, H_2_O_2_, ^•^OH, ^1^O_2_ and O_2_^•−^, being produced and/or eliminated in biological systems, play important roles in diverse normal biochemical functions and abnormal pathological processes^4^,^5^. Among them, HClO, generated from H_2_O_2_ and Cl^−^ by secreted myeloperoxidase (MPO) catalyzed in response to inflammatory stimuli *in vivo*^6^,^7^, plays a crucial role in the innate immune system. However, deregulation of HClO levels is implicated with many pathophysiological consequences including cardiovascular diseases, rheumatoid arthritis, and cancer^8^. Furthermore, it is reported that HClO may cause lysosomal rupture^9^, mitochondrial permeabilization^10^, proteinase inactivation^11^,^12^ and cell death through calcium dependent calpain^10^. Therefore, methods for sensitive and selective detection of hypochlorous acid/hypochlorite are of considerable significance for both disease diagnosis and exploration of its diverse pathophysiology^13^,^14^,^15^.

Several analytical methods such as HPLC^16^, electrochemical detection^17^, electrophoresis^18^, ultraviolet spectrophotometry^18^ and fluorescent probes^19^,^20^ have been used for the detection of hypochlorous acid. Among these reported methods, fluorescent probes are extensively employed for HClO detection and imaging *in vitro* and *in vivo* owing to their distinct advantages^21^. Recently, transition metal complexes have attracted a great of interest in the field of luminescent probes and cellular chemosensors due to their desirable chemical and photophysical properties, such as good water solubility, high chemical and photostability, intense polarized luminescence, visible-light absorption and emission, large Stokes shifts, long lifetimes and low cytotoxicity^22^,^23^,^24^,^25^. Among these transition metal complexes, ruthenium(II) complexes with three diimine ligands, such as 2,2′-bipyridine (bpy), 1,10-phenanthroline (phen), and bathophenanthroline derivatives are one type of potential candidates for environmental and biological HClO probing. Some ruthenium(II) complex based luminescent probes for hypochlorous acid have been developed^25^,^26^,^27^,^28^,^29^,^30^,^31^. These probes were generally designed based on the conjugation of the ruthenium complex with a HClO recognizing moiety, such as nitrophenyl derivatives^26^,^27^,^28^, phenothiazine^30^, ferrocene^31^ and oxime derivatives^25^. Recently, we found that the azo with an *o*-amino group could also be a candidate moiety for the recognition of HClO. As far as we know, there is no luminescence probe based on an azo-*o*-amino ruthenium(II) complex reported for the detection of HClO in aqueous solution^32^,^33^,^34^,^35^,^36^,^37^,^38^,^39^,^40^,^41^,^42^,^43^.

It is noted that the mononuclear ruthenium complex RuMAZO can detect Cu^2+^ without interference of HClO in HEPES (4-(2-hydroxyethyl)-1-piperazineethanesulfonic acid) buffer (Figure S1)^44^. Interestingly, this reaction could also be triggered by HClO in PBS (phosphate) buffer. However, the RuMAZO exhibited a relative low sensitivity to HClO and the reaction can be somehow interfered by Cu^2+^ (Figure S2b, S3). In our previous work^45^,^46^, a series of bimetallic ruthenium complexes were developed and some synergistic enhancing effect can be reached by the co-existing two ruthenium moieties, which can be quite helpful to solve the above mentioned problem. To continue our research and figure out this, another ruthenium moiety was introduced by incorporating the azo-phenanthroline derivative ligand into the ruthenium(II)-1,10-phenanthroline complex, to design a more sensitive and selective HClO probe and eliminate the interference of Cu^2+^. As shown in Fig. 1, the almost non-emissive dinuclear ruthenium(II) complex Ruazo formed highly luminescent product triazole-ruthenium(II) complex Rutazo in presence of hypochlorous acid with an oxidative cyclization of the azo and amino group in the dinuclear ruthenium(II) complex, which can detect HClO without interference of Cu^2+^ in the PBS buffer (Figure S2, S4), and showed highly sensitive and selective luminescent responses towards HClO without interferences of other ROS/RNS. Based on these features, we use this probe to investigate its luminescence response behavior towards exogenous HClO in living cells and living mouse successfully.

## Results

Firstly, the dynamics luminescence response of the probe to HClO was investigated in a PBS buffer (10 mM, pH 7.4) at room temperature. After the addition of HClO to the solution of Ruazo (10 μM), luminescence enhancement was clearly evident up in 3 min (Figure S5), then no further significant changes occurred, which suggests that the optimal reaction time for HClO detection for this probe is around 3 min. Thus, all of the UV–vis absorption and luminescence properties were investigated under the same conditions. As shown in Figure S6, the probe showed typical absorption spectra of the ruthenium(II)-1,10-phenanthroline complexes. The absorption band at 263 nm of the complexes is dominated by the π−π* transition of the ligands. While the absorption bands in the visible region (445 nm, 456 nm) are attributed to the metal-to-ligand charge transfer (MLCT) transitions for ruthenium(II) complexes. After reacting with HClO, the absorption between 490 nm and 550 nm caused by the absorption of phen-AZO ligand in the probe disappeared, the ligand absorption around 263 nm decreased at the same time.

**Figure 2:**
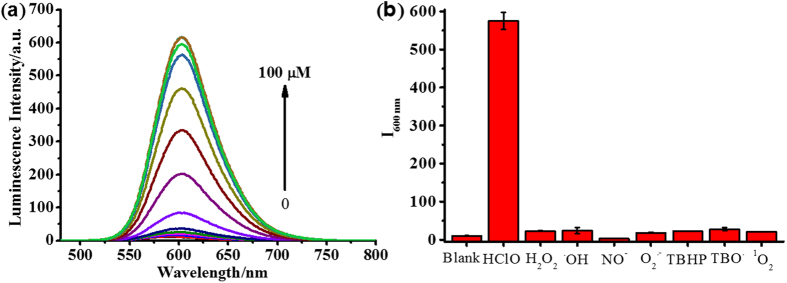
The Luminescence studies of Ruazo towards HClO. (**a**) Luminescence intensity of Ruazo (10 μM) with various concentrations of NaClO (0, 0.5, 3, 7, 10, 20, 30, 40, 50, 60, 70, 80, 90, 100 μM) in a PBS buffer (10 mM, pH 7.4). (**b**) Luminescence changes of Ruazo (10 μM) upon the addition of various ROS (100 μM) or RNS (100 μM) in a PBS buffer (10 mM, pH 7.4).

To investigate the sensitivity, selectivity of Ruazo to HClO, luminescence of the probe was measured with various concentrations of HClO. As shown in Fig. 2a, the emission intensity of Ruazo without HClO was negligible. With the addition of HClO from 0 to 80 μM, the emission intensity at 600 nm increased to over 50 folds upon excitation at 465 nm. Moreover, the logarithm of the luminescence intensity followed a good linear relationship with the concentration of HClO over the range of 0.5–50 μM (Figure S7) and the limit of detection (LOD) for HClO was determined to be 4.37 × 10^−7 ^M.

The specificity measurement of Ruazo with HClO was also investigated in 10 mM phosphate buffer of pH 7.4. As shown in Fig. 2b, after treated with various ROS and RNS (100 μM), the changes of the emission intensity of Ruazo (10 μM) were negligible in the presence of other ROS/RNS, such as H_2_O_2_, ^•^OH, NO^•^, O_2_^•−^, TBHP, TBO^•^ and ^1^O_2_. Whereas the emission of Ruazo treated with HClO (100 μM) resulted in highly luminescent signals. As potential interfering factors for specific detection of HClO, some common cations (100 μM), anions (100 μM) or amino acids (100 μM) were also examined under the same conditions (Figure S8). As expected, no obvious emission intensity changes were observed upon the additions of other cations, anions or amino acids. All of these demonstrated that the probe is highly specific for the detection of HClO.

Furthermore, the influence of pH on the emission intensities of the probe Ruazo and its titration with HClO was examined under different pH value (Figure S9). Neither the probe nor its reaction mixture with HClO had obvious signal changes over the pH range of 5–9. These results confirmed that the luminescence of Ruazo and its reaction with HClO were essentially pH-insensitive in the pH range of 5–9 and expected to work well under physiological conditions.

## Discussion

The mechanism of Ruazo in HClO detection was investigated. The UV–vis absorption changes after the addition of HClO indicated the structure change in Ruazo (Figure S6). All these were attributed to the oxidative cyclization of the quencher azo and amino group converted into luminescent benzotriazole by HClO, which was confirmed by ESI-MS and ^1^H NMR (Figure S10, 11).

**Figure 3:**
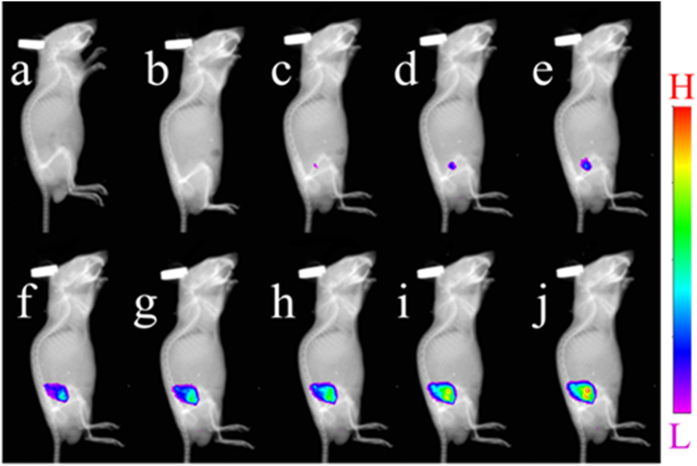
The luminescence images of live mice. Mice were given a subcutaneous injection of (**a**) PBS buffer (100 μL, 10 mM, pH 7.4), (**b**) 10 nmol Ruazo (100 μL in a PBS buffer (10 mM, pH 7.4)), (**c–j**) 10 nmol Ruazo (100 μL in a PBS buffer (10 mM, pH 7.4)) and then 80 nmol HClO in solution (50 μL) after 0, 5, 10, 20, 30, 40, 50 and 60 min, respectively.

The practical applicability of Ruazo for imaging HClO was investigated in living cells. HeLa cells were incubated with Ruazo (10 μM) exhibited not any obvious luminescence (Figure S12b). However, the cells incubated with HClO (50 μM) showed red luminescence (Figure S12e). The luminescence images at different concentrations of HClO (0, 20, 30, 40, 50, 60 μM) were also taken. As shown in Figure S13, the luminescence intensity enhanced with the increasing concentration of HClO. The results revealed that Ruazo can be used as an off-on luminescent probe for sensing HClO in living cells. The cytotoxicity of Ruazo to the HeLa cell lines was investigated with an MTT (3-(4,5-dimethylthiazol-2-yl)-2,5-diphenyltetrazolium bromide) assay after a 24 h treatment. The result showed that Ruazo exhibited no obvious cytotoxicity to the cells (Figure S14). Finally, luminescence imaging in mouse model was evaluated by taking advantages of excellent behavior of Ruazo towards HClO. As the control, the mice were given hypodermic injections of 10 nmol Ruazo (100 μL in a PBS buffer solution (10 mM, pH 7.4)) or a PBS buffer solution (100 μL, 10 mM, pH 7.4) into right backs respectively. Then, 80 nmol HClO in solution (50 μL) was injected subcutaneously after 5 min. Pictures were taken under the imaging system after the HClO were incubated for 0, 5, 10, 20, 30, 40, 50 and 60 min, respectively. As shown in Fig. 3, luminescence intensities of the region with Ruazo and HClO injected became stronger and stranger within 60 min. While almost no emission signals exhibited in the control group injected with Ruazo or PBS buffer alone. Thus, Ruazo is able to detect HClO through luminescent signal *in vivo*. Taken together, Ruazo can be a desired imaging agent for visualizing HClO *in vivo*.

In summary, a water-soluble dinuclear ruthenium(II) complex was developed as a luminescent probe for the detection of HClO. The weakly luminescent Ruazo can specifically and sensitively react with HClO in aqueous media and exhibit highly luminescence signal after the oxidative cyclization of the azo and *o*-amino group in the probe. Luminescent imaging detections for HClO were successfully achieved in living mouse. This hypochlorous acid induced oxidative cyclization reaction can be employed as a new route for HClO detection.

## Methods

All chemical reagents were purchased from commercial suppliers and used as received. All the organic solvents were analytical grade. Deionized water was used for all the measurements. ^1^H NMR and ^13^C NMR spectra were recorded on a Bruker 500 AVANCE III spectrometer with chemical shifts reported in ppm at room temperature (500 MHz for ^1^H NMR and 125 MHz for ^13^C NMR, Germany). Mass spectra were obtained with Thermo Fisher LCQ Fleet mass spectrometer (USA) or a LC/Q-Tof MS spectrometry (USA).

All spectrographic measurements were performed in 10 mM PBS buffer (pH 7.4). The pH of the testing systems was determined by a PHS-3C pH Meter (China). Absorption spectra were measured with a Shimadzu UV-1750 UV-vis spectrometer (Japan). Luminescence spectra were collected by using a Shimadzu RF-5301 fluorescence spectrometer (Japan). The mouse imaging experiment was conducted by Kodak *in-vivo* imaging system FX Pro (USA). Images of Hela cells were performed on an Olympus FV1000 confocal microscope (Japan).

All of the experiments were performed in compliance with the relevant laws and institutional guidelines, and were approved by Northwest A&F University.

The concentration determination and/or preparation of HClO and other reactive oxygen species (H_2_O_2_, ^•^OH, NO^•^, O_2_^•−^, ^1^O_2_, TBO^•^, TBHP) were made according to the literature^47^,^48^.

The synthetic route of Ruazo is shown in Figure S15. 5-amino-1, 10-phenanthroline is prepared according to the literature previously reported^49^. The azo-phenanthroline ligand was prepared through the coupling reaction of 5-amino-1,10-phenanthroline with its diazonium salt. The ruthenium(II) complex was obtained in a satisfactory yield (85%) through direct reaction of the azo-phenanthroline ligand with the appropriate molar ratios of *cis*-[Ru(phen)_2_Cl_2_] in ethanol.

### Phen-AZO

5-amino-1, 10-phenanthroline (195 mg, 1.0 mmol) was dissolved in 1.5 mL concentrated hydrochloric acid, then 4.0 mL cold solution of NaNO_2_ (69 mg, 1.0 mmol) was added. After stirred for 1 h under 0 °C, the mixture was added drop wise to 18.0 mL 5-amino-1, 10-phenanthroline (195 mg, 1.0 mmol) acetate buffer (1.5 g sodium acetate and 3.0 mL acetate) in 30 min. After the addition was completed, the mixture was stirred for 2 days. Then ammonia aqueous was added to this mixture to adjust to pH = 7 and then filtered, washed with pure water, purified by column chromatography on silica gel with CH_2_Cl_2_/CH_3_OH. Deep red solid was obtained, dried under vacuum with a yield of 308 mg, 77%. ^1^H NMR (500 MHz, TFA-*d*): δ (ppm) 10.18 (d, *J* = 8.5 Hz, 1H), 10.01 (d, *J* = 8.5 Hz, 1H), 9.58 (d, *J* = 4.0 Hz, 1H), 9.45 (t, *J* = 6.5 Hz, 2H), 9.39 (d, *J* = 4.5 Hz, 1H), 9.25 (d, *J* = 8.5 Hz, 1H), 9.09 (d, *J* = 5.5 Hz, 1H), 8.75 (s, 1H), 8.60 (dd, *J* = 8.5, 5.0 Hz, 1H), 8.49 (dd, *J* = 8.5, 5.5 Hz, 1H), 8.39 (dd, *J* = 8.5, 5.5 Hz, 1H), 8.23 (dd, *J* = 8.5, 4.5 Hz, 1H). ^13^C NMR (125 MHz, TFA-*d*): δ (ppm) 152.48, 148.69, 147.92, 146.97, 145.53, 142.19, 140.99, 140.92, 139.09, 138.90, 135.82, 133.64, 133.41, 132.57, 132.17, 130.87, 129.19, 127.01, 126.85, 126.06, 123.99, 122.83. ESI-MS: [M+H]^+^, calculated for [C_24_H_16_N_7_], 402.14, found: 402.13.

### Ruazo

*cis*-[Ru(phen)_2_Cl_2_]•2H_2_O (228 mg, 0.4 mmol) and phen-AZO (81 mg, 0.2 mmol) were dissolved in 30 mL ethanol, then the mixture was protected with nitrogen and refluxed for 12 h. The mixture was concentrated under reduced to about 2 mL. The residue was dropped to NH_4_PF_6_ solution and stirred for 30 min. Red precipitate was filtered and washed with cold water, dried under vacuum. Yield: 324 mg, 85%. ^1^H NMR (500 MHz, Acetone-*d*_*6*_): δ (ppm) 9.53 (d, *J* = 8.5 Hz, 1H), 9.40 (d, *J* = 8.5 Hz, 1H), 9.24 (d, *J* = 8.5 Hz, 1H), 8.91 (d, *J* = 8.5 Hz, 1H), 8.87–8.77 (m, 9H), 8.63–8.59 (m, 1H), 8.59–8.56 (m, 1H), 8.56–8.50 (m, 4H), 8.47–8.41 (m, 12H), 8.39 (dd, *J* = 5.0, 1.1 Hz, 1H), 8.14 (d, *J* = 4.5 Hz, 1H), 7.96–7.79 (m, 11H), 7.73 (dd, *J* = 8.5, 5.0 Hz, 1H). ^13^C NMR (125 MHz, Acetone-*d*_*6*_): δ (ppm) 154.99, 153.56, 153.34, 153.12, 152.97, 149.48, 148.86, 148.10, 143.25, 138.30, 137.02, 133.30, 133.06, 131.17, 130.96, 128.51, 128.24, 127.07, 126.47, 126.33, 126.26, 125.84, 113.02. HRMS: [M-2PF_6_^−^]^2+^, calculated for [C_72_H_47_F_12_N_15_P_2_Ru_2_], 807.5749, found: 807.5754.

### Rutazo

Ruazo (48 mg, 0.025 mmol) was dissolved in 10 mL acetonitrile and water (2/3, V/V), then sodium hypochlorite solution (0.25 mmol) was dropped to the mixture. Then the mixture was stirred for 5 min, concentrated and purified by chromatography to get red orange solid 41 mg, 86%. ^1^H NMR (500 MHz, CD_3_CN) δ (ppm) 9.41–9.31 (m, 2H), 9.08 (d, *J* = 7.5 Hz, 3H), 8.81 (d, *J* = 7.5 Hz, 1H), 8.70–8.62 (m, 8H), 8.35–8.25 (m, 7H), 8.24–8.17 (m, 5H), 8.14–8.09 (m, 3H), 8.08–8.01 (m, 4H), 7.80–7.63 (m, 12H). ESI-MS: [M-2PF_6_^−^]^2+^, calculated for [C_72_H_45_F_12_N_15_P_2_Ru_2_], 806.56, found: 806.47.

## Additional Information

**How to cite this article**: Liu, Z. *et al*. A dinuclear ruthenium(II) complex as turn-on luminescent probe for hypochlorous acid and its application for in vivo imaging. *Sci. Rep*. **6**, 29065; doi: 10.1038/srep29065 (2016).

## Supplementary Material

Supplementary Information

## Figures and Tables

**Figure 1:**
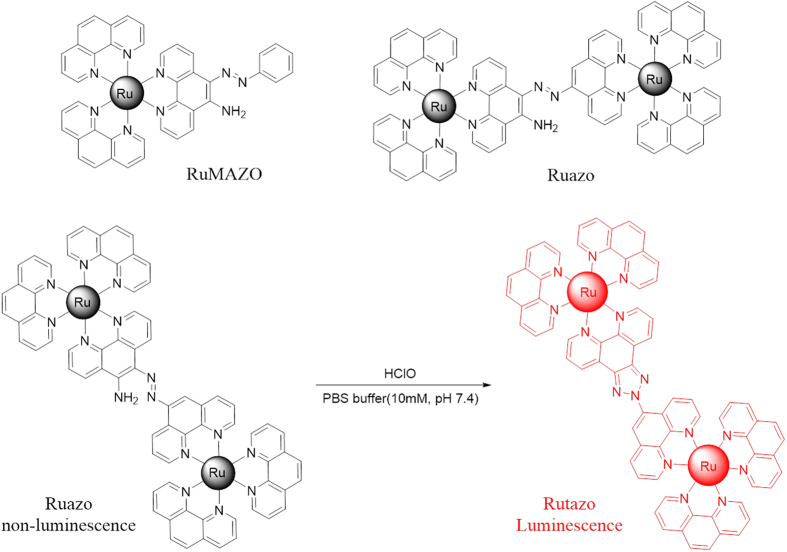
Molecular structures of RuMAZO and Ruazo. The proposed mechanism of the probe towards hypochlorous acid.
