# Supplementary material for: A dinuclear ruthenium(II) complex as turn-on luminescent probe for hypochlorous acid and its application for in vivo imaging
Source: Sci Rep. 2016 Jun 30;6:29065. doi: 10.1038/srep29065 (PMC4928191; doi:10.1038/srep29065)
Supplement: Supplementary Information [file srep29065-s1.pdf]

A dinuclear ruthenium(II) complex as turn-on luminescent probe for hypochlorous acid and its application for in vivo imaging

Zonglun Liu[1], Kuo Gao[2], Beng Wang[1], Hui Yan[1], Panfei Xing[1], Chongmin Zhong[1], Yongqian Xu[1], Hongjuan Li[1], Jianxin Chen[2], Wei Wang[2] & Shiguo Sun[1]

[1]College of Science, Northwest A&F University, Yangling, Shaanxi, 712100, China.

[2]Beijing University of Chinese Medicine, No.11 Beisanhuandonglu, Chaoyang District, Beijing, 100029, China.

Corresponding author

Correspondence to: Shiguo Sun, sunsg@nwsuaf.edu.cn; Jianxin Chen, cjx@bucm.edu.cn; Wei Wang, wangwei@bucm.edu.cn.

Calculation of the detection limit

The limit of detection (LOD) was calculated according to the formula in the literature. (*Spectroscopy* 2003, 12, 112-114. *Anal. Chem.* 2009, 11, 4555-4559. *Dalton Trans.* 2013, 42, 15113-15119).

$$\mathrm{LOD} = \frac{3\sigma}{\mathrm{k}}$$

Where σ is the standard deviation of the blank solution measured by 10 times; k is the slope of the calibration curve.

The detection limit was calculated based on the fluorescence titration. Increasing amounts of HClO were added to the probe (10 μM) in PBS buffer. Representation of fluorescence at the appropriate wavelength vs. concentration of HClO allowed the limit of detection to be calculated. From Figure S7, we get slope k = 0.0313, and σ value is 0.00456. Thus using the formula we get the limit of detection (LOD) = 4.37×10^{-7} M.

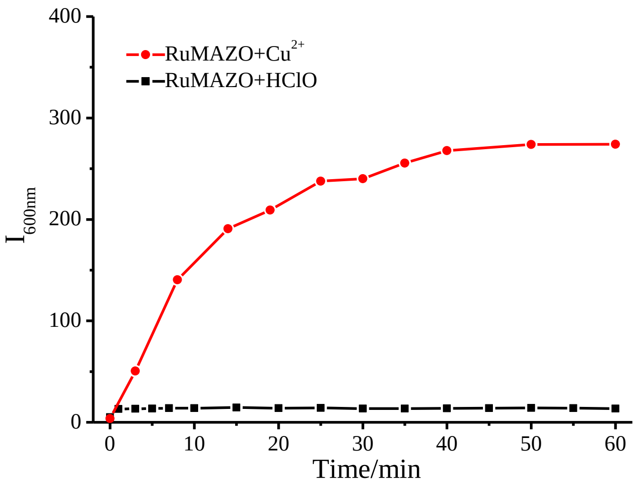

Figure S1. Time-dependent luminescence intensity of RuMAZO (10 μM) at 600 nm treated with HClO (100 μM) or Cu^{2+} (20 μM) in HEPES buffer (10 mM, pH 7.4) with λ_{ex} = 465 nm.

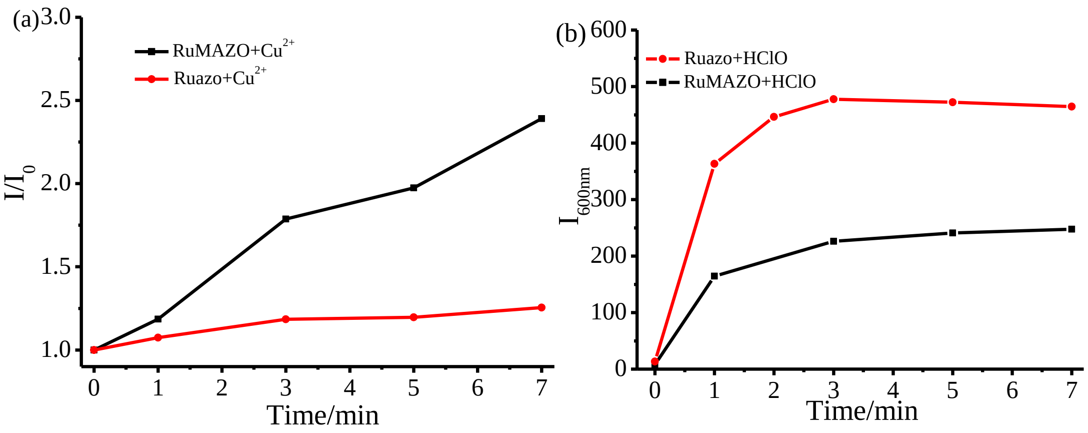

Figure S2. (a) Time-dependent luminescence changes of RuMAZO (10 μM) or Ruazo (10 μM) treated with Cu^{2+} (100 μM) in PBS buffer (10 mM, pH 7.4). (b) Time-dependent luminescence intensity of Ruazo (10 μM) and RuMAZO (10 μM) treated with HClO (100 μM) in PBS buffer (10 mM, pH 7.4) with λ_{ex} = 465 nm, λ_{em}= 600 nm.

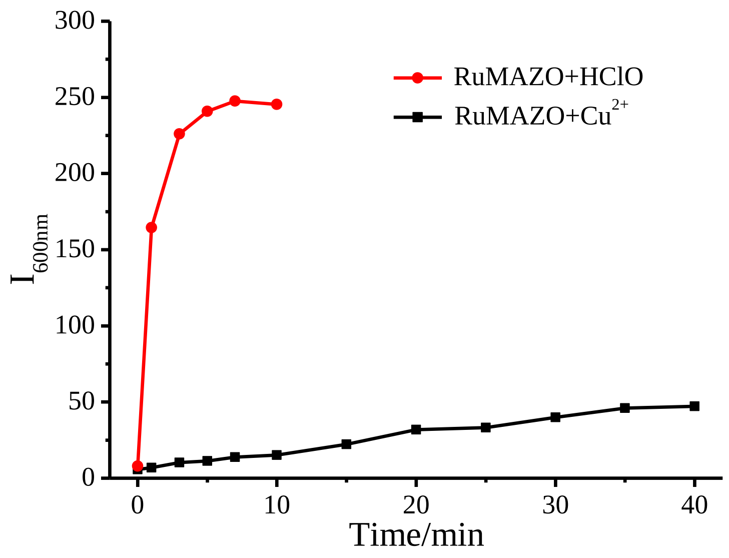

Figure S3 Time-dependent luminescence intensity of RuMAZO (10 μM) treated with HClO (100 μM) or Cu^{2+} (100 μM) in PBS buffer (10 mM, pH 7.4) with λ_{ex} = 465 nm, λ_{em}= 600 nm.

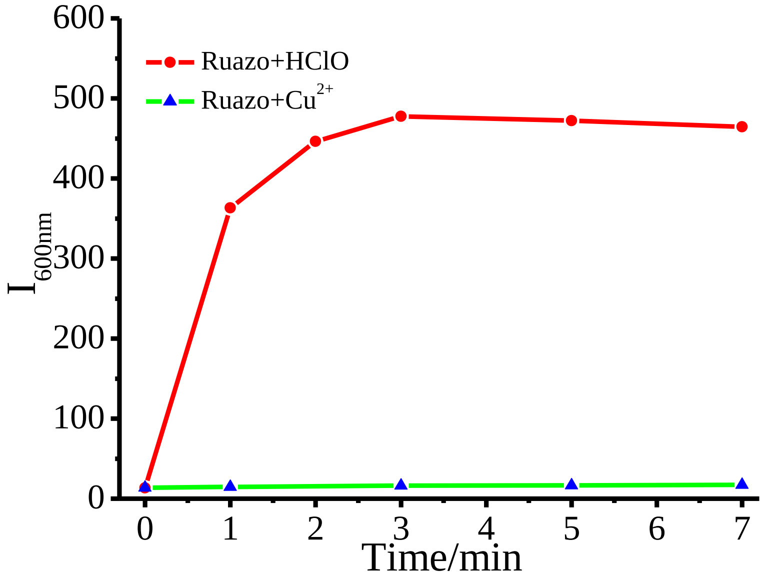

Figure S4 Time-dependent luminescence intensity of Ruazo (10 μM) treated with HClO (100 μM) or Cu^{2+} (100 μM) in PBS buffer (10 mM, pH 7.4) with λ_{ex} = 465 nm, λ_{em}= 600 nm.

Kinetics of luminescence enhancement profile

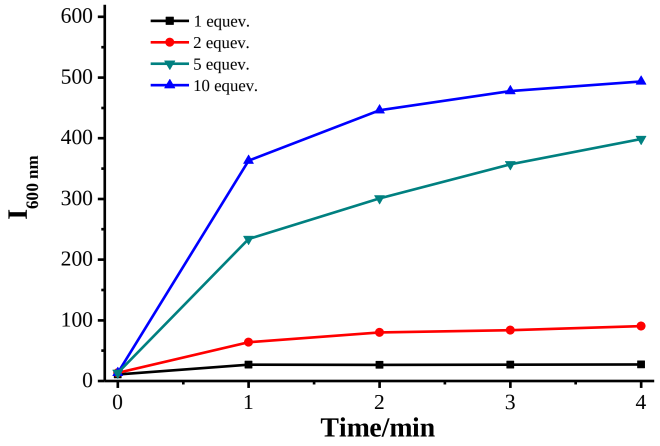

Figure S5. Time-dependent luminescence intensity of Ruazo (10 μM) at 600 nm treated with various concentrations of HClO in PBS buffer (10 mM, pH 7.4) with λ_{ex} = 465 nm, λ_{em} = 600 nm.

UV-Vis absorption spectra of the probe

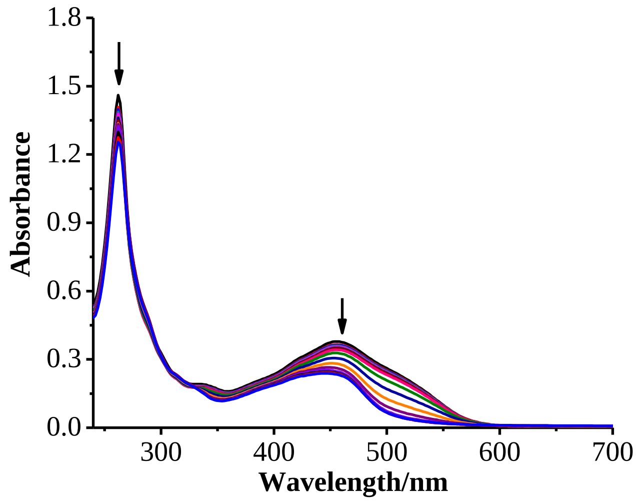

Figure S6. UV-Vis spectral changes of Ruazo (10 μM) upon the addition of various concentrations of HClO (0-100 μM) in a PBS buffer solution (10 mM, pH 7.4).

Luminescence spectra of the probe

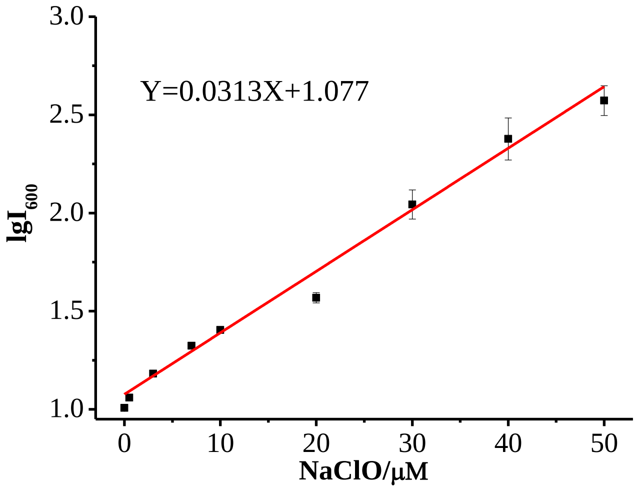

Figure S7. A linear correlation between the logarithm of the emission intensity of Ruazo at 600 nm and concentrations of HClO (0.5–50 μM).

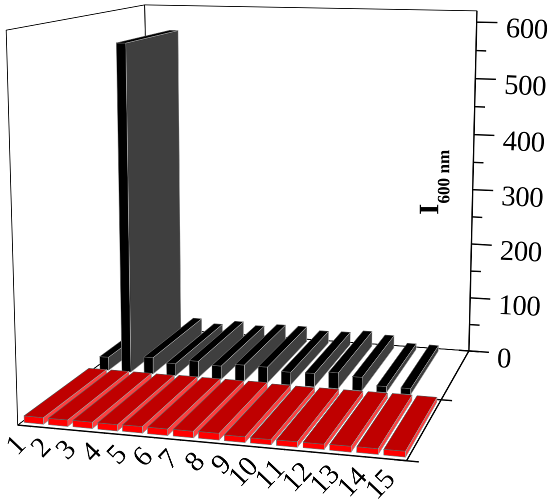

Figure S8. Luminescence changes of Ruazo (10 μM) upon the addition of various common cations (100 μM), anions (100 μM) or amino acids (100 μM) in PBS buffer solution (10 mM, pH 7.4). The black bars from 1-14 represent the luminescence responses toward Blank, HClO, AcO^{-}, HCO_3^{-}, S_2^{-}, SO_4^{2-}, NO_2^{-}, Cl^{-}, SO_3^{2-}, Cys, Hcy, GSH, Al^{3+}, Ba^{2+}; the red bars from 1-15 represent addition of various common cations (Ca^{2+}, Cd^{2+}, Cr^{3+}, Fe^{3+}, Hg^{2+}, K^{+}, Li^{+}, Mg^{2+}, Na^{+}, Ni^{2+}, Pb^{2+}, Zn^{2+}, Co^{2+}, Ag^{+}, Cu^{2+}).

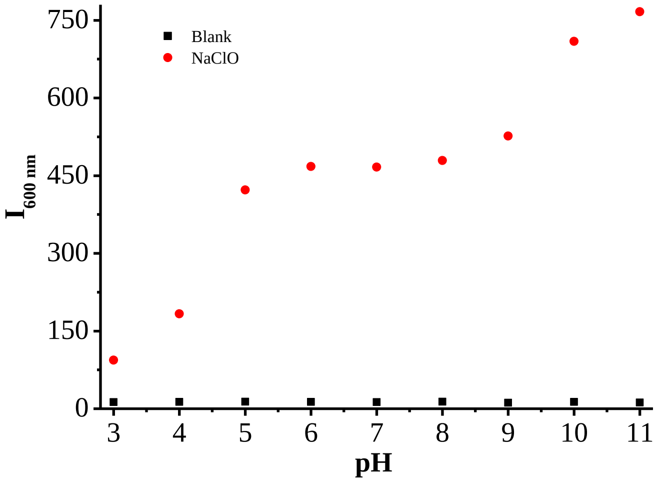

Figure S9. The luminescence intensity of Ruazo (10 μM) and its titration with HClO (100 μM) under different pH value (2-13). λ_{ex} = 465 nm, λ_{em} = 600 nm.

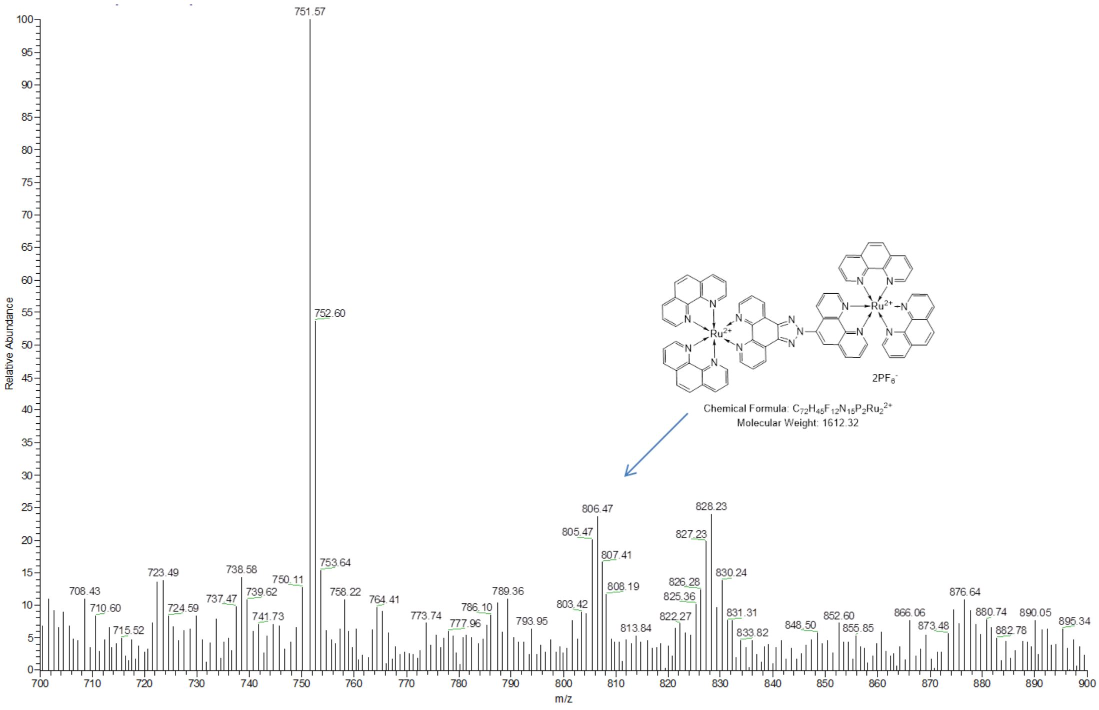

Figure S10. MS spectrum of the product the probe Ruazo reacted with HClO.

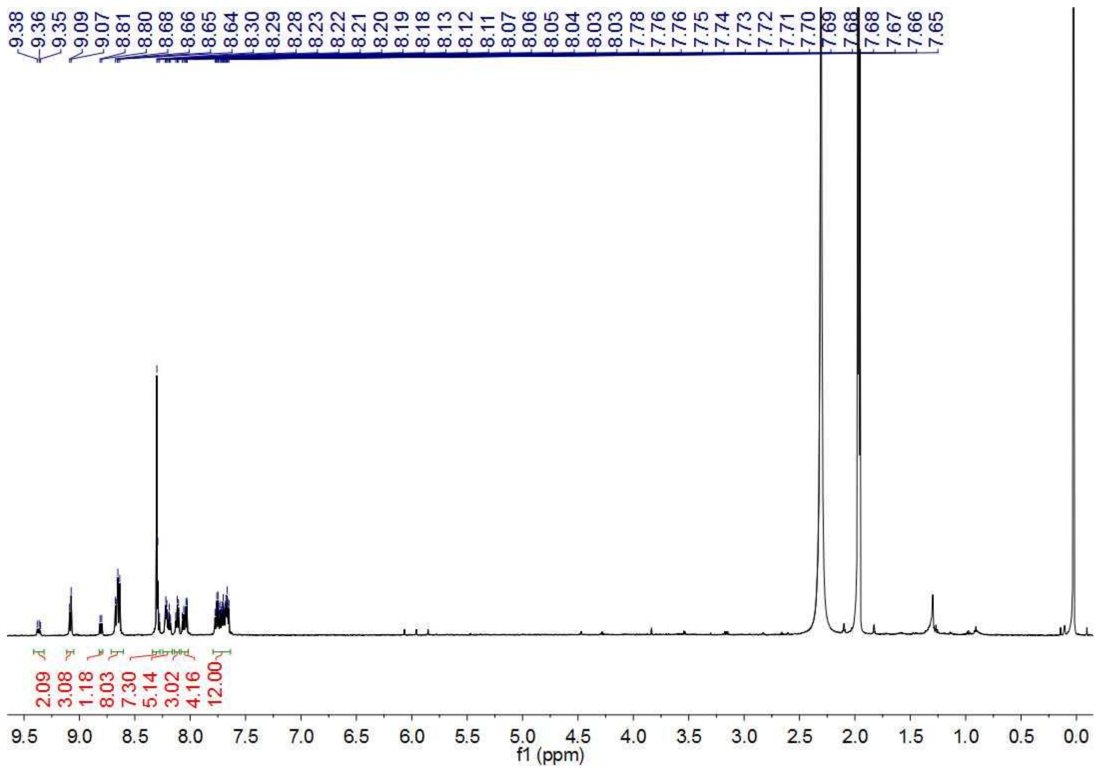

Figure S11. ^{1}H NMR spectrum of the Rutazo.

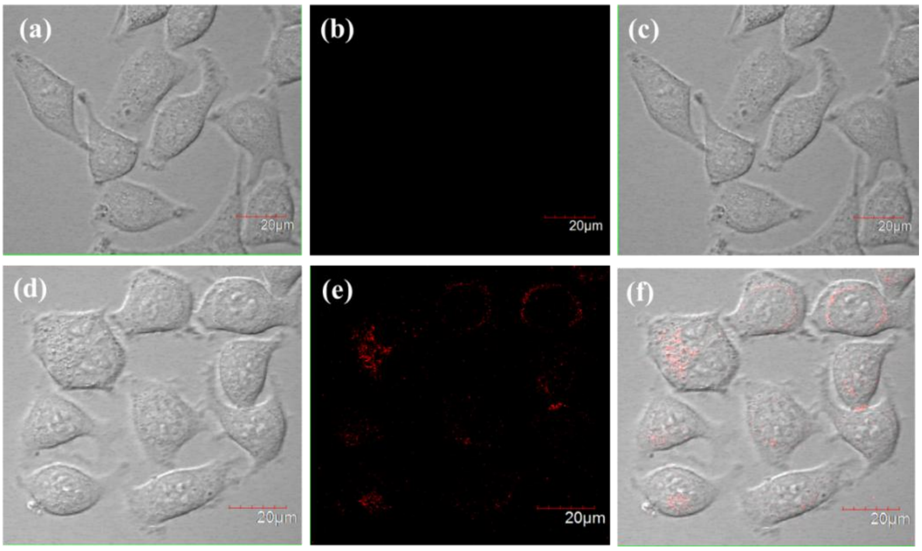

Figure S12. Confocal fluorescence images of HClO in HeLa cells. (a) Bright-field image of cells incubated with probe (10 μM) for 90 min; (d) Bright-field image of cells after treatment with 50 μM HClO for 10 min and then treatment with 10 μM probe for 90 min; (b) and (e) Luminescence -field images of the HeLa cells (550–650 nm); (c) and (f) Overlap of bright-field and luminescence. λex = 488 nm.

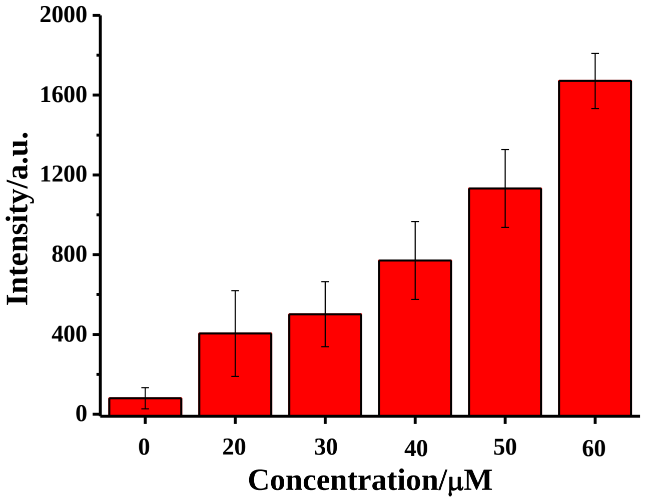

Figure S13 The luminescence intensity at different concentrations of HClO (0, 20, 30, 40, 50, 60 μM) and the concentration of Ruazo was 10 μM.

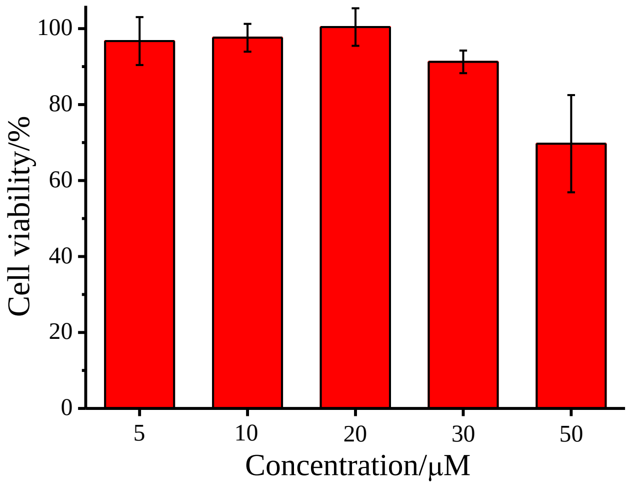

Figure S14. Percentage of HeLa cell viability remaining after cell treatment with Ruazo (the untreated cells were considered to have 100% survival).

Synthesis of Ruazo

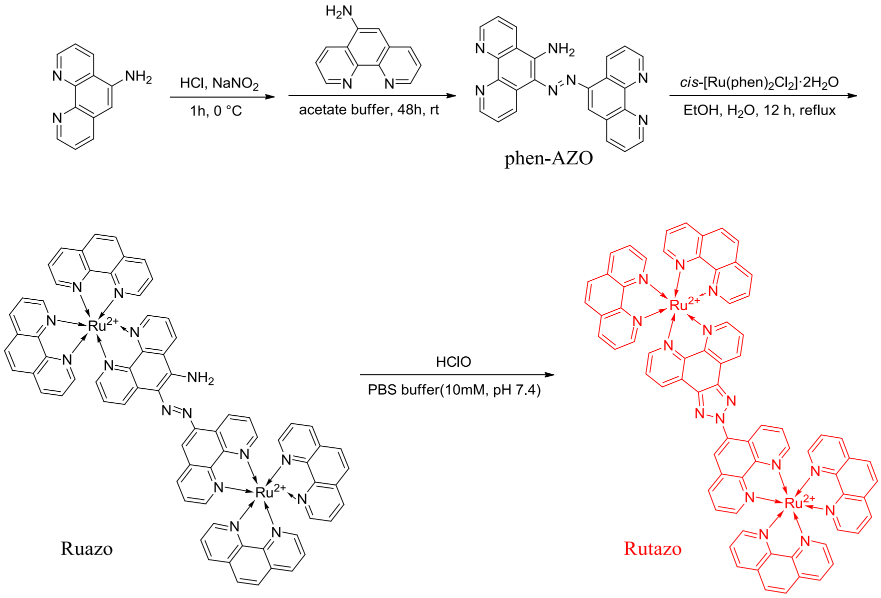

Figure S15. Synthesis of Ruazo and the proposed mechanism of response of the probe towards hypochlorous acid.

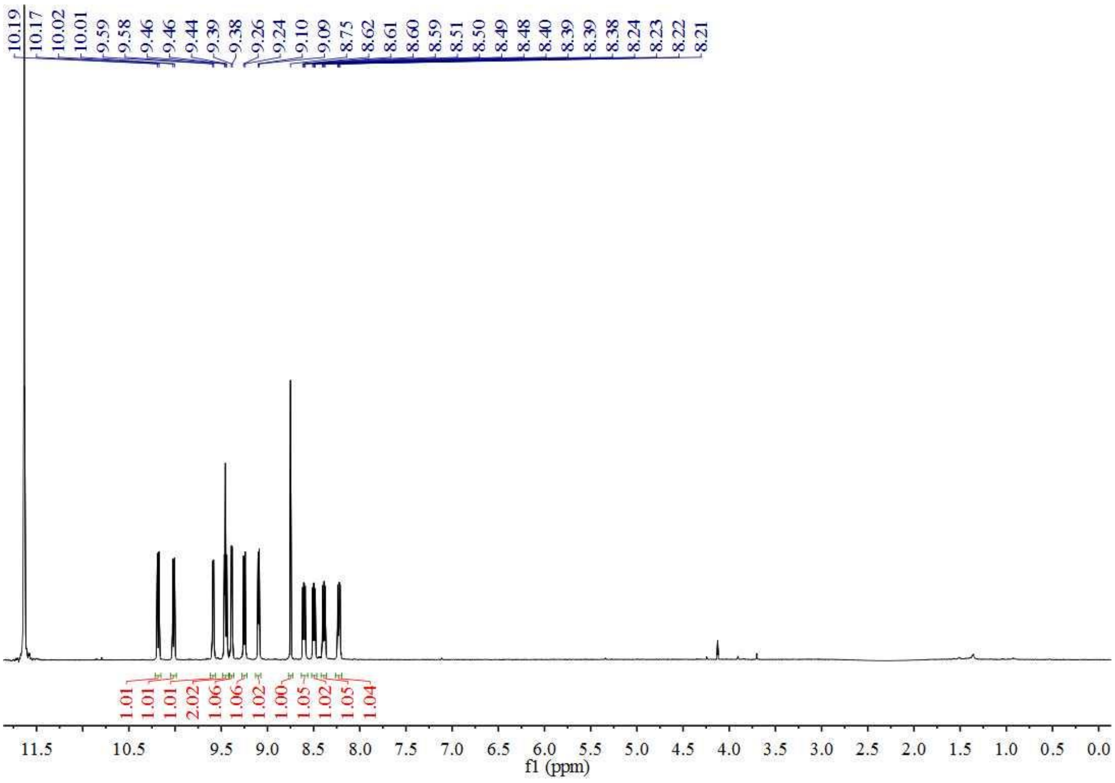

Figure S16. ^{1}H NMR spectrum of ligand phen-AZO.

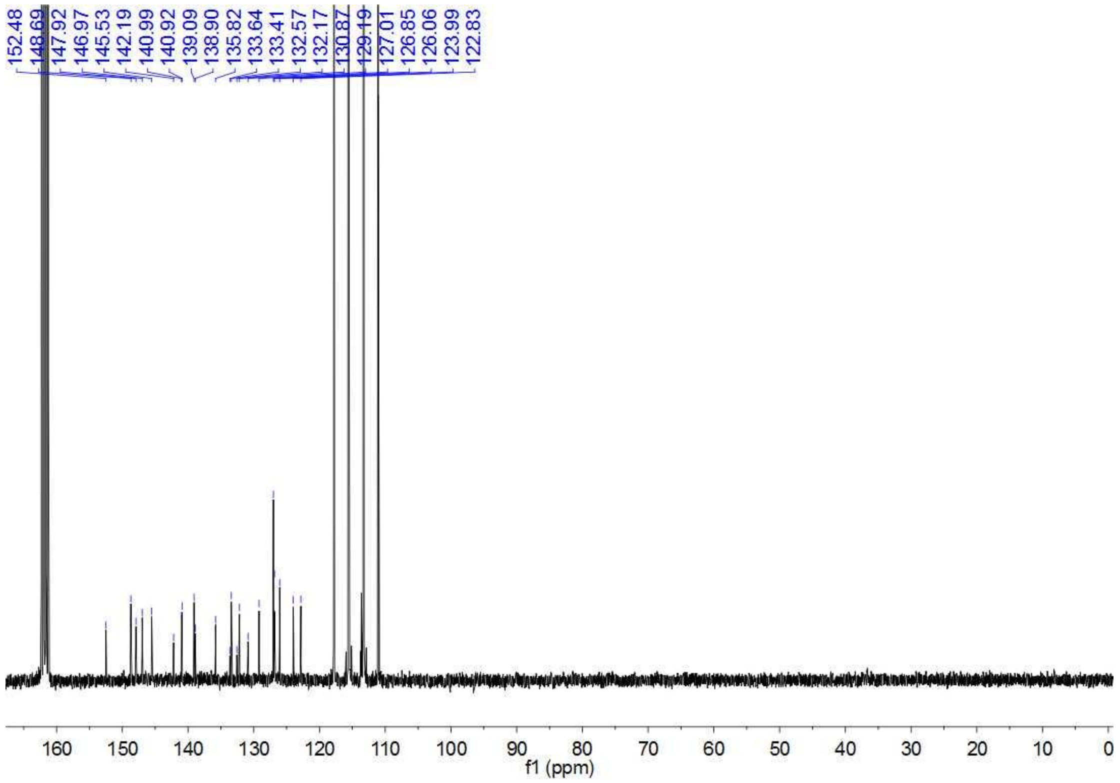

Figure S17. ^{13}C NMR spectrum of ligand phen-AZO.

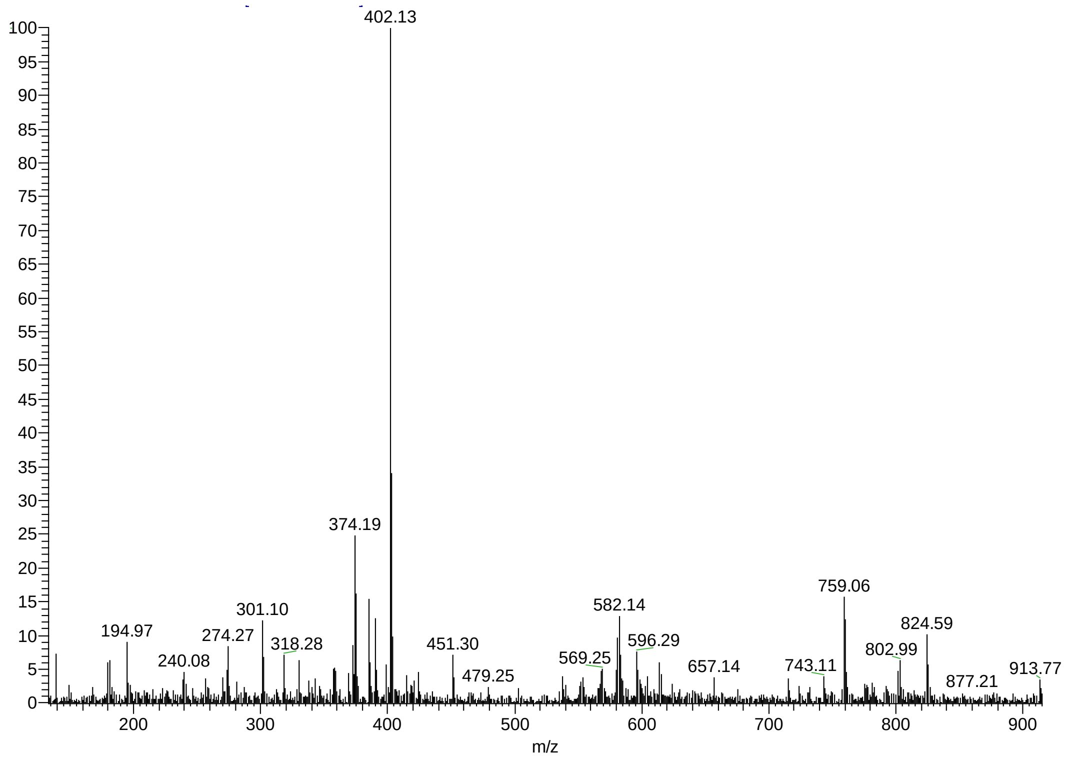

Figure S18. MS spectrum of ligand phen-AZO.

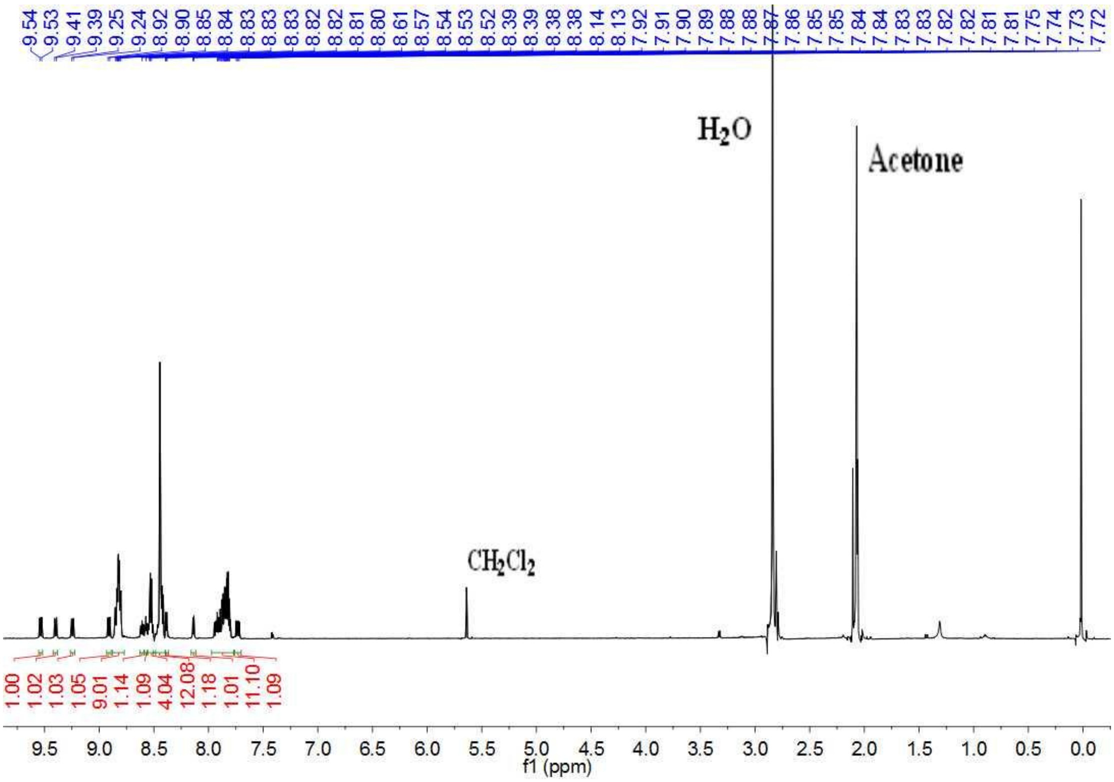

Figure S19. ^{1}H NMR spectrum of the probe Ruazo.

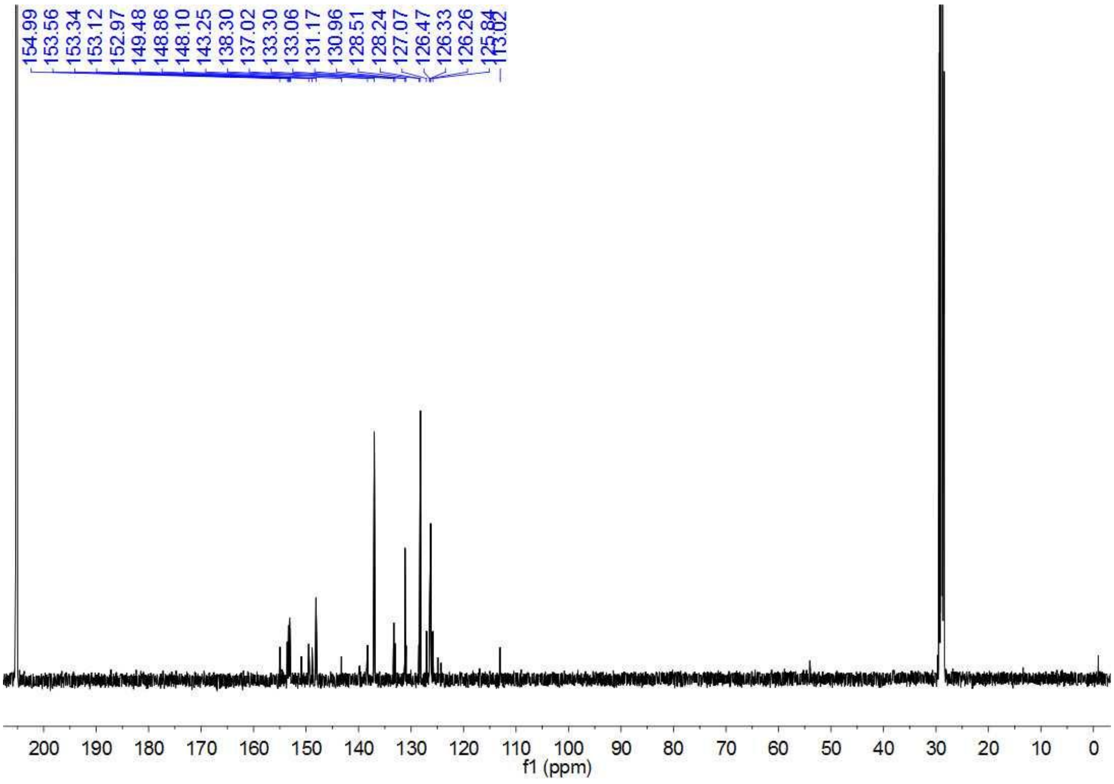

Figure S20. ^{13}C NMR spectrum of the probe Ruazo.

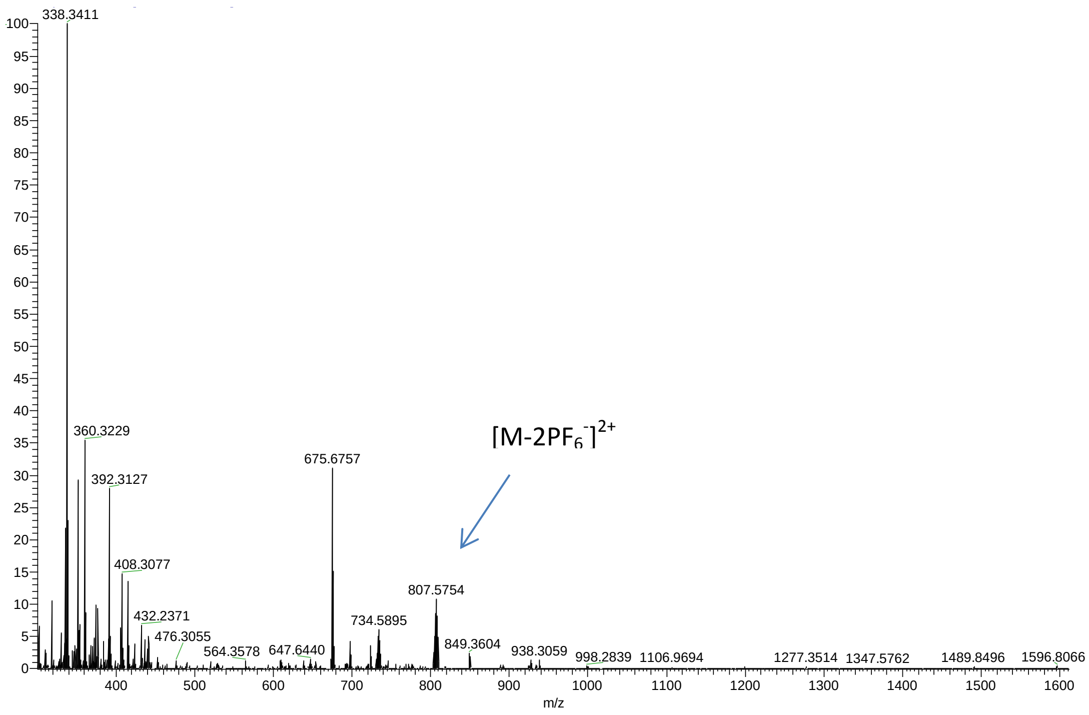

Figure S21. HRMS spectrum of the probe Ruazo.
